# Strength Development and Thermogravimetric Investigation of High-Volume Fly Ash Binders

**DOI:** 10.3390/ma12203344

**Published:** 2019-10-14

**Authors:** Zhiyuan Zhou, Massoud Sofi, Elisa Lumantarna, Rackel San Nicolas, Gideon Hadi Kusuma, Priyan Mendis

**Affiliations:** 1 Department of Infrastructure Engineering, The University of Melbourne, Parkville, VIC 3010, Australia; zhiyuan.zhou@unimelb.edu.au (Z.Z.); elu@unimelb.edu.au (E.L.); rackel.san@unimelb.edu.au (R.S.N.); pamendis@unimelb.edu.au (P.M.); 2 David Reid Homes, Glen Waverley, VIC 3150, Australia; gideonkusuma@drhme.com.au

**Keywords:** high volume fly ash, binder, heat of hydration, adiabatic temperature rise, chemically bound water (W_B_)

## Abstract

To address sustainability issues by facilitating the use of high-volume fly ash (HVFA) concrete in industry, this paper investigates the early age hydration properties of HVFA binders in concrete and the correlation between hydration properties and compressive strengths of the cement pastes. A new method of calculating the chemically bound water of HVFA binders was used and validated. Fly ash (FA) types used in this study were sourced from Indonesia and Australia for comparison. The water to binder (w/b) ratio was 0.4 and FA replacement levels were 40%, 50% and 60% by weight. Isothermal calorimetry tests were conducted to study the heat of hydration which was further converted to the adiabatic temperature rise. Thermo-gravimetric analysis (TGA) was employed to explore the chemically bound water (W_B_) of the binders. The results showed that Australian FA pastes had higher heat of hydration, adiabatic temperature rise, W_B_ and compressive strength compared to Indonesian FA pastes. The new method of calculating chemically bound water can be successfully applied to HVFA binders. Linear correlation could be found between the W_B_ and compressive strength.

## 1. Introduction 

Due to environmental problems worldwide such as climate change, sustainability issues have increasingly become a concern in the 21st century. In order to address the sustainability issues, various industries have taken actions to replace products or processes that is energy intensive [1,2]. The building material production is the third-largest CO_2_ emitting industry party in the world, mostly attributed to the manufacturing of concrete [3]. Ordinary Portland cement (OPC) is the major binder in concrete material and the production of OPC consumes massive energy while generating a large amount of CO_2_ [1,4]. Supplementary cementitious materials (SCMs) have been used as supplements or part replacement of OPC in construction projects [5]. 

Fly ash (FA) is one of the most popular SCMs and is the solid waste produced from coal combustion in electricity stations [6]. Use of FA as part replacement of OPC reduces the carbon footprint associated with OPC production whilst it turns the industrial waste into a value-added product. Apart from benefits to the environment, incorporating FA in concrete brings about numerous advantages to concrete material itself. Incorporation of FA in concrete at low dosages (less than 50% by weight of OPC) is beneficial to the long-term strength development typically after 28 days [5,7], due to the hydration of fly ash (pozzolanic reaction) generating denser hydration products C-S-H in concrete compared to OPC alone [8,9,10]. The interfacial transition zone (ITZ) between aggregates and cement can be enhanced by incorporating FA as the FA improves the packing of mixes as well as producing denser hydration products in the long term as mentioned above [10]. It also improves the concrete workability, mainly due to the round shape of FA particles contributing to better fluidity of concrete pastes [11,12]. Numerous studies have indicated better durability of concrete when FA is added because the hydration products of FA reduces pores in concrete [13,14]. It has also been found that use of FA reduces the heat of hydration at early ages and thus decreasing the possibility of thermal cracking associated with hydration heat [6,12,15]. 

Despite plenty of merits, the dosage of FA to replace cement in typical construction is limited to up to 40% by weight of total binder [16], mainly because of the lower rate of early age strength development in most projects [17]. The slow early age strength development of OPC concrete containing FA is associated with lower pozzolanic reaction of FA in comparison with OPC [18]. Apart from that, FA produced from different power plants generally have variability in physical and chemical properties which makes strength development difficult to predict [1]. Especially for pastes containing higher volume fly ash (HVFA) when fly ash replacement level of Portland cement is not less than 50% [19]. The mechanical properties of FA based concrete varies hence introducing uncertainties into the construction practice. Thus, it is important to assess the hydration of cement paste with FA from different regions to better understand the behavior of FA concrete.

Some commonly used techniques to assess the hydration degree of binder in concrete reported in the literature includes isothermal calorimetry and thermogravimetric analysis (TGA). Isothermal Calorimetry can conveniently monitor the hydration heat over a continuous time frame from the onset of the hydration [18,20]. It has also been used to measure heat release from cement paste with addition of FA [21,22]. Although heat released from pastes or mortar can be measured using calorimetry, it is difficult to correlate the released heat of the binder to that of concrete. This is primarily due to the presence of aggregates and heat dissipation rate of the system. There has been attempts to correlate rheological and mechanical properties of mortar to concrete [23]. Adiabatic temperature rise is calculated using measured isothermal calorimetry considering the temperature effects on hydration rate using an Arrhenius apparent activation energy approach [24,25]. Characterisation of the concrete exothermic reaction is directly related to the possibility of thermal cracking of mass concrete elements which is a concern in the construction industry [2].

For study of concrete hydration at ages after approximately 3 days from casting, isothermal calorimetry is not suitable as the heat produced could be too subtle to capture [21]. In this case, Thermogravimetric analysis (TGA) is often used to investigate the hydration of cement [21,26]. Several studies have considered the application of TGA to cement with FA addition [21,27,28]. TGA is instrumental in quantifying hydration reactions as different composition of cement hydrates can decompose in different temperature ranges during the heating process of TGA. Therefore, the chemically bound water (W_B_) at a certain age can be calculated from the mass loss of different compounds in pastes to indicate the degree of hydration. Bhatty [29], Pane et al. [21], and Monteagudo et al. [30] provided approaches based on TGA measurements to calculate W_B_ and the degree of hydration of binder materials (blended cement). Furthermore, Deboucha et al. [26] modified the aforementioned author’s methods to provide a more accurate way of calculation. The approach was successfully applied to cement with blast furnace slag and limestone fillers respectively. However, Deboucha et al.’s approach of calculating W_B_ from TGA has not been used for cement blended with FA. Zhang et al. [31] evaluated the hydration process of HVFA cement pastes (with FA replacement level of 40%, 50%, and 60%) by heating the pastes to find non-evaporable water content. It was found from the study that the non-evaporable water content is not a good indicator of degree of hydration for HVFA pastes as it does not necessarily increase over time. Thus, a proper method should be established to evaluate the hydration product of OPC pastes with HVFA.

This study proposes a new method developed from the study of Deboucha et al. [26] to calculate the chemically bound water (W_B_) of OPC pastes with HVFA. Two types of fly ash sourced from Australia and Indonesia are used for comparison. The hydration properties are investigated by isothermal calorimetry and thermogravimetric analysis (TGA). The accuracy of the proposed method of calculating W_B_ from TGA will be validated by comparing with isothermal calorimetry and compressive strength results. The method presented herein synthesises approaches based on traditional calculations proposed by previous researchers [21,26,29,30]. It is equally making modifications to increase the accuracy of estimating W_B_ for HVFA pastes.

## 2. Research Significance

Typically, a maximum of 30% to 40% of cement in structural concrete is replaced with FA to turn it into ‘green’ concrete which features less energy consumption and CO_2_ emission. Builders are often reluctant to consider replacing cement content with FA because of the reduction in concrete strength at early ages which, in turn, results in delays in construction schedule. Due to variability in physical properties and chemical composition of FA, it is a costly exercise to conduct full scale tests to evaluate engineering properties of FA based concrete. Alternative tests such as calorimetry and TGA provides indication of reaction kinetics of the blended mixes. This paper reports the results of investigation considering hydration properties of HVFA binders using FA from Indonesia and Australia. FAs from two different regions are considered for comparison and interest. Cement replacement levels of 40%–60% by FA is considered. The aim is to gain a better understanding of hydration kinetics and strength gain of concrete containing regional FAs. Isothermal calorimetry and TGA tests were conducted to quantify the hydration reaction of the mixes containing FA from different places. A new method of calculating the chemically bound water to correlate the hydration properties to compressive strength for cement pastes with high volume of FA was proposed and validated. 

### 2.1. Materials and Mix Design

The cement used in this research was type I Ordinary Portland cement (OPC) for general purpose supplied by Cement Australia. The FA used in experiment were Australian FA also supplied by Cement Australia and Indonesian FA from a steam-powered electric generator Paiton, East Java, Indonesia. The Chemical composition of OPC, Australian FA and Indonesian FA were provided in Table 1. 

Referring to ASTM C618 [32], both types of FA can be categorised as Class F FA as the conditions SiO_2_ + Al_2_O_3_ + Fe_2_O_3_ ≥ 70% and CaO < 10% are satisfied. The alkali content Na_2_O is less than 1% for type I OPC. Thus, the alkali content can be too minor to affect the fly ash reaction as several researches indicated that effective improvement on mechanical properties was obtained by the use of alkali largely more than 1% of the binder [16,33,34]. Additionally, the alkali in Indonesian FA and Australian FA are close to each other, thus the alkali content would not cause visible difference in hydration and strength of Indonesian and Australian FA pastes. The loss on ignition (LoI) of both FA are within the limit of 12% for class F fly ash by ASTM C618 [32]. 

**Figure 1:**
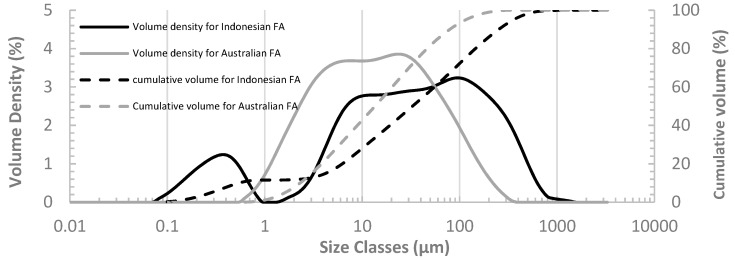
Particle size distribution for both Indonesian FA and Australian FA.

The particle size distribution for both Indonesian FA and Australian FA are plotted in Figure 1 for comparison. From Figure 1, it is noted that D_50_, representing the median particle size for Indonesian FA, is 31.7 μm and for Australian FA is 24.9 μm. It demonstrates that there are 50% of the Indonesian FA particles smaller than 31.7 μm while 50% of the Australian FA particles smaller than 24.9 μm. Australian FA has a larger quantity of small particles compared to Indonesian FA. The difference in particle size distribution of FA can affect the properties of OPC pastes containing FA. Several studies have indicated that OPC pastes containing FA with smaller particles can have higher reactivity and thus higher strength [34,35].

Cement pastes with FA replacement ratios including 0, 40%, 50% and 60% were adopted throughout the experimental work in this research to investigate the properties of HVFA pastes. All the experiment in this research including isothermal calorimetry, compressive strength testing, and TGA follow the mix design below in Table 2.

### 2.2. Isothermal Calorimetry

The early age hydration kinetics of cement paste containing FA were assessed by using a TAM Air 8-channel isothermal calorimeter. Immediately after manually mixing the cement pastes based on Table 2 for 5 min, about 30 g of each sample was put into an ampoule with a cap on and loaded into a channel of an isothermal calorimeter. With the temperature of the calorimeter kept at 23 °C, the measurement of calorimeter continued for 40 h to allow the recording of heat flow and cumulative heat of hydration over time from the beginning of the cement hydration. The recorded heat flow and cumulative heat were normalised with respect to the total mass of cementitious material of each sample for the comparison between different samples.

### 2.3. Converting of Heat of Hydration to Adiabatic Temperature Rise

Equation (1) correlates the heat of hydration to the adiabatic temperature rise in concrete:

(1) $$Q\left(t\right)=C_{c}\Delta T\left(t\right)\cdot \frac{m_{s}}{m_{c}}\;$$

where:

Q(t) = the cumulative heat released by concrete hydration at time t (kJ/kg)
$\Delta T\left(t\right)=T_{i}\left(t\right)-T_{0}$, and refers to the self-heating of concrete
$T_{i}\left(t\right)$ = the temperature of concrete at time t (°C)
$T_{0}$ = the initial temperature of concrete mix (°C)
$m_{S}$ = the mass of sample (kg)
$m_{c}$ = the mass of cement in the sample (kg)
$C_{c}$ = the specific heat of concrete (kJ/(kg K))

As the heat of hydration evolving over time was captured by the isothermal calorimetry test, Equation (1) could be transformed into Equation (2) as follows to determine the adiabatic temperature rise from the heat of hydration:

(2) $$\Delta T\left(t\right)=\frac{Q\left(t\right)}{C_{c}}\cdot \frac{m_{c}}{m_{s}}$$

From previous literature, the specific heat of cement paste was found to be within a small range from 916 to 920 kJ/(kg K) during the hydration process [36]. Bentz et al. [37] indicated that the change of proportion between OPC and FA would have little impact on the specific heat of pastes. In this study, $C_{c}$ was assumed to be 918 kJ/(kg K) in the calculations herein. 

### 2.4. Compressive Strength Testing

The compressive strength of paste samples following the mix design in Table 2 were tested at the age of 1, 3, 7, and 28 days. The samples were casted using cylindrical moulds with a diameter of 50 mm and height of 100 mm. After de-moulding, the samples were then cured in water tank at controlled room temperature of 23 °C until the designated time of testing. Technotest Modena Italy four column automatic testing machine (Model KE 300, Modena, Italy) was used for the testing. 

### 2.5. Thermo-Gravimetric Analysis (TGA)

TGA testing was conducted for FA paste samples at the age of 1, 3, 7, and 28 days. At the date of testing, small pieces of samples were collected from the samples crushed by compressive strength testing. The small pieces of samples were then grounded by using mortar and pestle until they were fine enough to pass 75 μm sieve completely. Afterwards, the powders passing the 75 μm sieve were collected to stop the hydration reaction. The powders were soaked entirely in acetone and then dried using an air pump. The process of soaking and drying was repeated for five times in order to ensure complete termination of hydration before the dried powders were subjected to TGA.

The TGA were performed using a PerkinElmer Diamond TG/DTA machine (Waltham, MA, USA) with 115 V under the air flow of 200.0 mL/min. After placing a sample into a platinum crucible in the machine, the heating program was set as:

1. Hold for 15.0 min at 40.00 °C;
2. Heat from 40.00 °C to 1000.00 °C at 10.00 °C/min;
3. Hold for 5.0 min at 1000.00 °C;
4. Cool from 1000.00 °C to 30.00 °C at 30.00 °C/min.

Decomposition of cement hydrates occurs during TGA testing and can be generally divided into three major stages [26]. The first stage is associated with the loss of free water in the temperature range of 25 °C to 105 °C and the loss of water from hydrates (dehydration) from 105 °C to 400 °C. The second stage links to the dehydroxylation of Portlandite from 400 °C to 600 °C. The third stage corresponds to decarbonation of CaCO_3_ from 600 °C to 800 °C. However, the temperature ranges for different stages varies a little bit for different authors [26]. In this research, the following temperature boundaries in Table 3 is considered. 

The chemically bound water (W_B_) can be obtained by using TGA test to record the mass loss of decomposition of cement samples at different stages. Bhatty [29] indicated that the W_B_ could be calculated conforming to following equation:

(3) $$W_{B}=Ldh+Ldx+0.41\left(Ldc\right)$$

where the 0.41 is used as the conversion factor to obtain the bound water from the decomposition of CaCO_3_.

Pane et al. proposed another approach of calculating W_B_ as follows [21]:

(4) $$W_{B}=Ldh+Ldx+\left(Ldc-Ldc_{a}\right)$$

where $Ldc_{a}$ is the mass loss caused by the decomposition of CaCO_3_ during TGA test on anhydrous samples [26]. It is recorded as the mass difference between 600 °C and 780 °C. 

Monteagudo et al. [30] combined the conversion factor 0.41 and the mass loss $Ldc_{a}$ and suggested the equation below:

(5) $$W_{B}=Ldh+Ldx+0.41\left(Ldc-Ldc_{a}\right)$$

Furthermore, Deboucha et al., [26] incorporated all contributions from the authors mentioned above and made corrections by subtracting the loss on ignition of both OPC and mineral additives to obtain the equations below:

(6) $$W_{B}=Ldh+Ldx+0.41\left(Ldc-Ldc_{a}\right)-\left(m_{c}\times LOI_{c}+m_{A}\times LOI_{A}\right)+m_{d}$$

(7) $$m_{c}=\frac{m_{sample}-m_{B}\times \left(x+\frac{W}{B}\right)}{1+LOI_{c}}$$

(8) $$m_{A}=\frac{m_{sample}-m_{B}\times \left(\left(1-x\right)+\frac{W}{B}\right)}{1+LOI_{A}}$$

where:

$m_{c}$ and $m_{A}$ are the mass of cement and mineral additives respectively, normalised with respect to the initial sample mass as presented in Equations (7) and (8).
$LOI_{c}$ and $LOI_{A}$ are the loss on ignition of OPC and mineral additive, respectively:
$m_{B}$ is the mass of binder
x is the replacement ratio of cement by mineral additive
$\frac{W}{B}$ is the water to binder ratio
$m_{d}$ is the device’s drift defined as the weight gain of the empty crucible when subject to a high temperature. It is ignored here as it is not significant.

In this research, Equation (6) was adopted to calculate the chemically bound water $W_{B}$ of the cement samples. For the ease of comparison between different mixes, $W_{B}$ of each sample was then normalized with respect to the sample mass to give the $W_{B}$ in per gram of each sample.

## 3. Results and Discussion

### 3.1. Early Age Heat of Hydration Development

**Figure 2:**
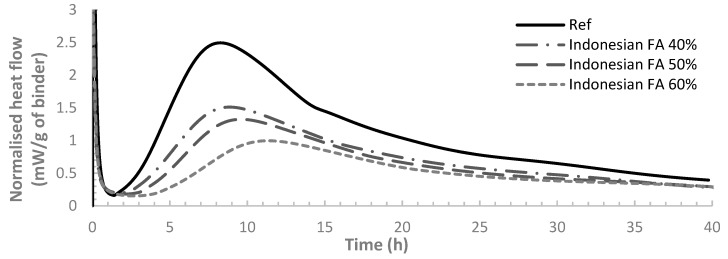
Normalised heat flow for Indonesian FA and OPC pastes.

**Figure 3:**
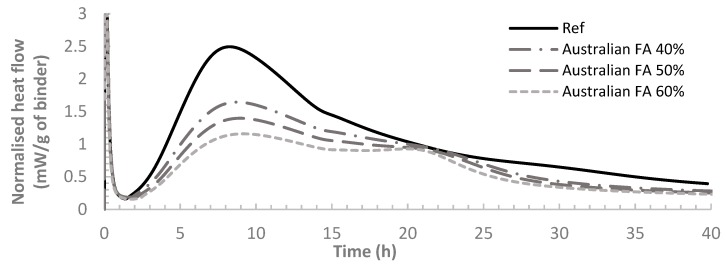
Normalised heat flow for Australian FA and OPC pastes.

Figure 2 and Figure 3 demonstrate the heat flow per gram of each binder sample over 40 hours from the start of cement hydration for binders with Indonesian and Australian FA respectively. From both graphs, the results show that the heat flow curve shifted to the right with the incorporation of FA. With the FA replacement ratio of OPC increased from 40% to 60%, the extent of the shift became larger, indicating a larger retarding effect on the hydration by FA. The observed trend has been reported by previous studies which have also demonstrated that the rate of cement hydration would be decelerated by FA with higher FA content contributing to larger decelerating effect [21,36,37,38]. It was also found that class F FA can start to hydrate after a week or even more from the time of mixing. The slower reaction rate is mainly attributed to the delayed FA reaction, as FA particles break down only after OPC hydrates for a while until hydration products of OPC accumulates to reach a PH of 13.2. Some research also indicated that FA could almost be considered as dormant fillers at very early age [39].

Three peaks can be observed on the heat flow curve (Figure 2 and Figure 3). The first peak is very high, representing the dissolution of the surface of cement particles. The hydration at this stage mainly involves C_3_A [2] (Tri-calcium aluminate, 3CaO·Al_2_O_3_). After a short duration of this period, a dormant period of about 1–2 hours follows featuring a very low hydration rate. The concrete is still workable during the dormant period. The second peak then came after the dormant period, indicating the hydration after breaking down of the surface layer of cement particles. 

Both Figure 2 and Figure 3 present that the second peak occurs later for the mixes with higher FA dosage, showing a larger retardation of the hydration reaction. The second peak of the heat flow profile was found to be linked to the setting of cement. To quantify the retardation of cement setting, the setting time of all the pastes, recorded as the time at which the second peak of heat flow occurs, are present in Table 4 along with the delay of setting time for all the FA pastes in comparison to OPC. From Table 4, it can also be seen that for both Australian FA pastes and Indonesian FA pastes, the setting time is longer for pastes with higher FA dosages. For the same FA replacement ratio, pastes with Australian FA tend to have a smaller retarding time of setting compared to pastes with Indonesian FA. Therefore, pastes containing Australian FA have higher reaction rate and thus more rapid setting compared to Indonesian FA at comparative ratios of replacement. This could be due to the size effect of fly ash. Comparing the particle size distribution of Indonesian FA and Australian FA (Figure 1), although Indonesian FA has a higher content of small particles than the Australian FA, the median value of particle size distribution (D_50_) of Australian FA particles is smaller than that of Indonesian FA. The smaller particles in blended cement can serve as nucleation sites to accelerate cement hydration. Since Australian FA has overall smaller particle size (smaller D_50_) compared to Indonesian FA, there could be more nucleation sites in Australian FA pastes for cement hydration. 

**Figure 4:**
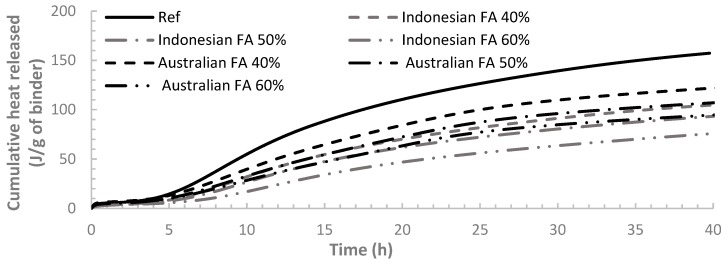
Normalised cumulative heat released for all the cement paste samples.

The third peak of heat flow curve corresponds to the renewed hydration of C_3_A and the formation of ettringite by consuming gypsum. It is shown in Figure 4 that, for the binders containing FA, the third peak of heat flow curve became more noticeable compared to the reference case (pure OPC sample). Higher FA dosage result in a higher third peak. The same finding was reported by Beart et al. [40] which also stated that the third peak of the heat flow curve can be more conspicuous in HVFA paste. It is mainly due to the nucleation sites offered by FA to accelerate the ettringite formation [31,41,42]. However, Figure 2 and Figure 3 indicate that the third peak of the heat flow curves for binders containing Indonesian FA is not as remarkable as Australian FA, indicating a less intense reaction. This could also be the reflection of the overall smaller particle sizes of Australian FA compared to Indonesian FA. 

The normalised cumulative heat for all the samples is presented in Figure 4. It is illustrated that the heat released over time for samples containing FA is visibly smaller than the pure OPC sample (reference case) at early age. The heat of hydration also decreased with the increase in FA replacement level indicating the delayed hydration rate of cement with FA replacement. It can also be seen that binders containing Australian FA generate higher hydration heat than binders containing Indonesian FA for the same FA dosage. This is not surprising as it has been found from heat flow curves that the reaction rate of pastes containing Australian FA is higher than Indonesian FA at very early age. Higher reaction rate can consequently result in higher cumulative heat. Other researches also validated that cement pastes with finer FA particles can have higher heat of hydration [43,44].

### 3.2. Adiabatic Temperature Development

**Figure 5:**
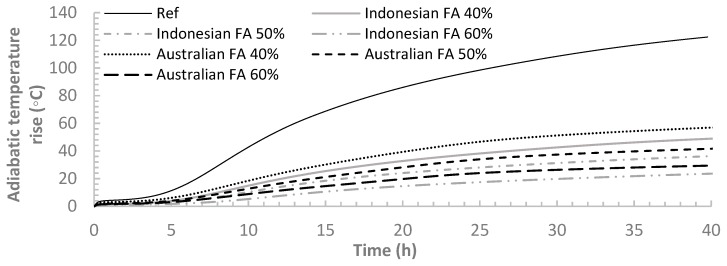
Adiabatic temperature rise for all the paste samples.

The adiabatic temperature rises for all the samples calculated from calorimetry results are presented in Figure 5. The results clearly showed that at a w/c ratio of 0.4, increasing FA replacement ratio of cement from 40% to 60% resulted in decreasing temperature rise at early age. These results are in good agreement with the cumulative hydration heat results from isothermal calorimetry. The incorporation of FA has beneficial effects on the hydration process by reducing the adiabatic temperature rise. Several previous researches showed the effectiveness of Class F FA in reducing the adiabatic temperature rise [5]. Therefore, the incorporation of FA could be instrumental in mitigating the temperature differential in mass concrete structure like dams where high temperature tends to occur due to cement hydration. It was also reported in another study that the adiabatic temperature decreases with the increase of FA dosage at different w/c ratio of either 0.53 or 0.25 with the increase of FA dosage when FA dosage is not less than 40% [29]. 

Comparing binders containing Australian FA and Indonesian FA, the binders containing Australian FA had higher temperature rise than those with Indonesian FA. This is not surprising due to the higher hydration rate of binders containing Australian FA. Binders with Australian FA has more small particles thus more nucleation sites compared to Indonesian FA.

### 3.3. TGA Results

The results for TGA including the calculated values of Ldh, Ldx, Ldc and W_B_ for all the mixes are presented in Table 5. The TG curves (A1–A6) for the calculations are provided in the Appendix. 

**Figure 6:**
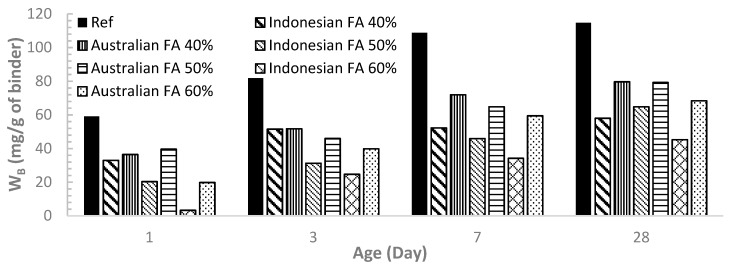
Comparison of W_B_ between Indonesian FA and Australian FA pastes.

**Figure 7:**
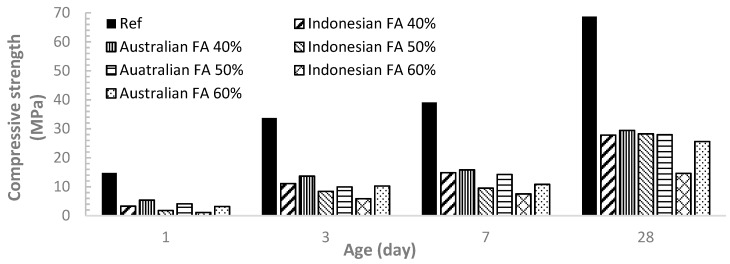
Comparison of compressive strength between Indonesian FA and Australian FA pastes.

The chemically bound water content (W_B_, presented as mg per gram of the original binder sample) as an indication of the degree of hydration for all the mixes are presented in Figure 6 and Figure 7 for comparison. 

It can be seen from Figure 6 that for both Australian FA and Indonesian FA pastes, W_B_ generally increases with the curing time, indicating increasing degree of hydration over time. For pastes at the same ages, the incorporation of FA decreased W_B_, consequently decreasing the degree of hydration in comparison to OPC (the reference case). W_B_ continued to decrease as the FA dosage increases from 40% to 60%.

Figure 6 also shows that under the same FA replacement level, Australian FA pastes had higher W_B_ meaning more content of hydration products than Indonesian FA at each age of 1, 3, 7 and 28 days. This result matches with the isothermal calorimetry finding. As Australian FA has more percentage of smaller particles than Indonesian FA, Australian FA pastes can have higher rate of hydration and more formation of hydration products compared to Indonesian FA. 

### 3.4. Compressive Strength Test Results 

Figure 7 demonstrate the compressive strength development for all the samples at ages of 1, 3, 7 and 28 days. The compressive strengths for all the pastes increased over time due to the hydration process. Incorporation of FA at 40%, 50% and 60% significantly reduces the compressive strength in comparison to the OPC paste (reference case). Although it was found that FA can contribute to the strength by pozzolanic reaction at the age of 28 days [9], the strength development for both Australian FA pastes and Indonesian FA pastes shows that the FA replacement level up to 40% can reduce more than 50% of the strength compared to OPC. For both Indonesian FA and Australian FA pastes tested at each age level, the compressive strength decreased with the increase of FA replacement ratio from 40% to 60%. 

The results also showed that Australian FA pastes tended to have a slightly higher compressive strength than the Indonesian FA paste at each FA replacement level. This finding was expected as calorimetry and TGA results indicated higher hydration rate and consequently higher degree of hydration for Australian FA pastes at early ages up to 28 days. This result matches with Erdoğdu and Türker study. They indicated that mortars incorporating FA with particle sizes less below 45 μm can have strength higher than mortars with original ash with all fractions while FA with particles sizes above 45 μm can have less strength [10]. In this experiment Australian FA and Indonesian FA has 79% and 58% FA below 45 μm respectively. Thus more percentage of FA below 45 μm could contribute to more strength gain. Other researches also indicated higher strength for cement pastes containing FA with finer particles [9,12]. 

### 3.5. Correlation between Compressive Strength and W_B_

**Figure 8:**
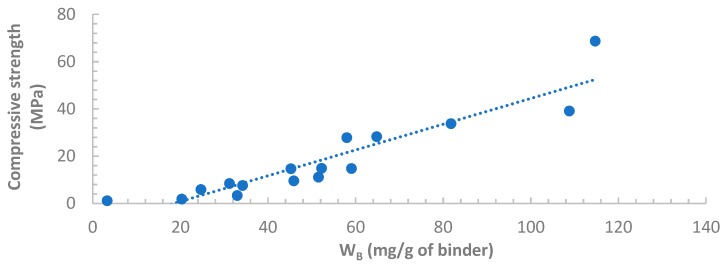
Compressive strength vs. W_B_ for OPC and Indonesian FA pastes.

**Figure 9:**
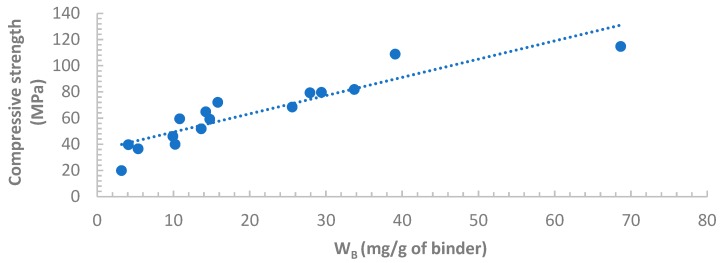
Compressive strength vs. water bound (W_B_) for OPC and Australian FA pastes.

The compressive strengths of all samples are plotted against the associated chemically bound water W_B_ in Figure 8 and Figure 9. Good correlations between the compressive strength and W_B_ can be observed for both Indonesian FA and Australian FA pastes. This result validated the approach developed from Deboucha et al.’s study to calculate W_B_ of HVFA cement pastes. Additionally, W_B_ of HVFA binders obtained from this method can potentially serve as a predictive tool for the compressive strength development at early ages as satisfactory correlations were found. 

## 4. Concluding Remarks

This study compared the cement pastes containing high volume of fly ash sourced from Indonesia and Australia regarding hydration and strength properties. A new method of calculating the chemically bound water (W_B_) of high-volume fly ash pastes from thermogravimetric analysis was also validated. This method considers the loss on ignition of cementitious materials and decarbonation of anhydrous materials based on traditional calculation approaches, in order to increase the accuracy of the calculation. The conclusions were drawn as follows. 

The method of calculating W_B_ developed from Deboucha et al.’s study can be properly applied to HVFA pastes. For both Indonesian and Australian fly ash cement pastes, the calculated W_B_ was linearly correlated with compressive strength. There could be a potential of predicting compressive strength of HVFA binders from the W_B_ obtained from TGA tests. 

As Australian FA has overall finer particle size and more percentage of particles below 45 μm than Indonesian FA, Australian FA pastes had higher heat of hydration and adiabatic temperature rise compared to Indonesian FA pastes at the same replacement level of FA. 

Australian FA pastes with finer particles of FA had more formation of hydration products indicated by higher W_B_ content thus higher compressive strength compared to Indonesian FA pastes at the same replacement level of FA. 

## Figures and Tables

**Table 1:** The chemical composition of type I ordinary Portland cement (OPC), Australian fly ash (FA) and Indonesian FA used in this research.

		Type I OPC	Australian FA	Indonesian FA
Composition (%)	SO₃	2.7	0.3	0.21
	CaO	63.7	6.2	9.24
	SiO₂	19.9	56.2	45.67
	Al₂O₃	4.6	23	21.85
	Fe₂O₃	2.57	7.9	15.73
	MgO	1.39	1.5	3.51
	K₂O	0.69	0.95	1.34
	Na₂O	0.09	0.79	0.52
	P₂O₅	0.04	1.2	0.28
	Mn₂O₃	0.06	<0.1	0.17
	Cr₂O₃	0	min	min
	SrO	0.07	min	min
	Na_2_O	0.5	0.79	0.52
	LoI	3.9	0.9	11.31
Mineral Phase (%)	C₃S	65.78		
	C₃A	7.82		
	C₄AF	7.88		

**Table 2:** Mix design for experiment in this research (Note: FA/b represents the ratio of fly ash to total binder by weight).

Mix	w/b	FA/b
Ref	0.4	0
Indonesian FA 40%	0.4	0.4
Indonesian FA 50%	0.4	0.5
Indonesian FA 60%	0.4	0.6
Australian FA 40%	0.4	0.4
Australian FA 50%	0.4	0.5
Australian FA 60%	0.4	0.6

**Table 3:** Temperature boundaries for decomposition phases of cement pastes considered in thermo-gravimetric analysis (TGA) test [24].

Decomposition Phase	Temperature Boundaries (°C) [26]
Dehydration (Ldh)	105–400
Dehydroxylation (Ldx)	400–600
Decarbonation (Ldc)	600–1000

**Table 4:** Setting time and delay of setting estimated from isothermal calorimetry for blended cement compared with OPC.

	Setting Time (h)	Delay of Setting (h)
Reference mix	8.26	0
Indonesian FA 40%	8.84	0.58
Indonesian FA 50%	9.71	1.45
Indonesian FA 60%	11.26	3.00
Australian FA 40%	8.76	0.50
Australian FA 50%	9.00	0.75
Australian FA 60%	9.20	0.94

**Table 5:** Calculated Ldh, Ldx, Ldc, Ldca and W_B_ for all the mixes.

Mix	Age (Day)	m_sample (mg)	Ldh (mg)	Ldx (mg)	Ldc (mg)	Ldca (mg)	W_B_ (mg/g)
Ref	1	55.83	4.18	1.32	2.41	2.13	59.08
	3	47.87	12.59	0.10	2.29	2.02	81.80
	7	50.90	5.80	1.94	1.98	1.62	108.81
	28	38.87	4.28	1.34	2.46	1.63	114.72
Indonesian FA 40%	1	56.63	2.48	1.29	2.56	2.62	32.99
	3	56.51	3.50	0.56	3.38	3.29	39.17
	7	49.09	3.65	0.94	3.23	3.11	61.43
	28	46.62	3.61	0.65	2.48	2.50	58.00
Indonesian FA 50%	1	56.98	2.15	1.26	2.64	2.59	20.34
	3	45.26	2.26	0.91	2.25	2.15	31.23
	7	52.24	3.31	1.15	2.75	2.73	45.91
	28	50.78	4.23	1.03	2.58	2.46	64.83
Indonesian FA 60%	1	61.22	1.49	1.59	1.96	2.08	3.29
	3	55.13	2.54	1.41	2.50	2.57	24.70
	7	54.32	3.07	1.28	2.44	2.38	34.21
	28	47.28	3.16	1.13	1.88	1.79	45.24
Australian FA 40%	1	44.45	1.91	0.43	1.72	1.72	36.50
	3	47.04	2.64	0.69	1.19	1.52	51.74
	7	42.91	2.98	0.59	1.11	0.58	71.95
	28	40.98	3.50	0.70	2.00	1.85	79.64
Australian FA 50%	1	48.40	2.27	0.49	2.12	48.40	39.64
	3	48.99	2.41	0.64	1.71	48.99	46.02
	7	39.41	2.21	0.83	1.77	39.41	64.79
	28	34.48	3.18	0.53	0.39	34.48	79.28
Australian FA 60%	1	51.96	1.80	0.31	1.73	1.73	19.81
	3	41.74	2.08	0.40	2.05	1.94	39.86
	7	46.08	2.62	0.76	2.36	1.58	59.44
	28	42.69	3.50	0.72	1.63	1.43	68.40

**Figure A1:**
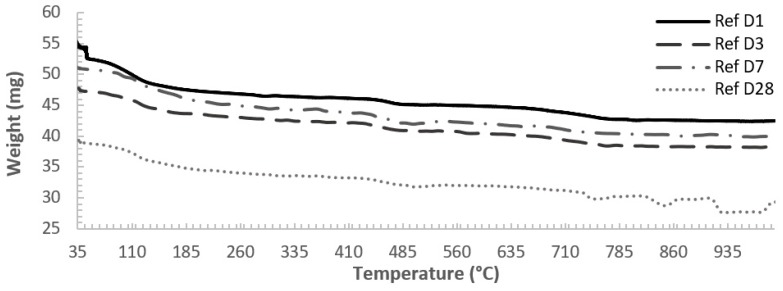
The TG curves for OPC paste at ages of 1, 3, 7 and 28 days.

**Figure A2:**
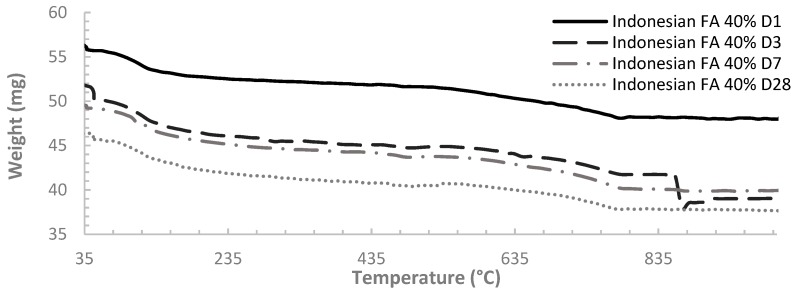
The TG curves for 40% Indonesian fly ash paste at ages of 1, 3, 7 and 28 days.

**Figure A3:**
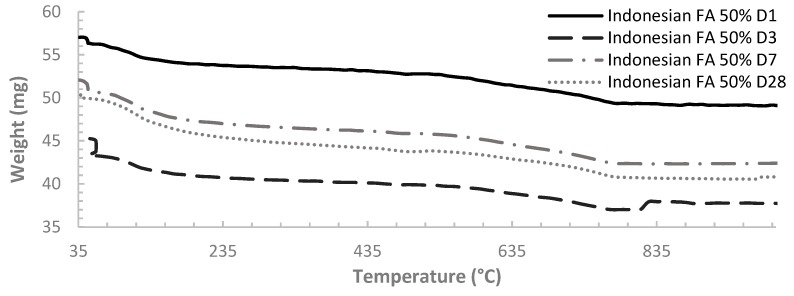
The TG curves for 50% Indonesian fly ash paste at ages of 1, 3, 7 and 28 days.

**Figure A4:**
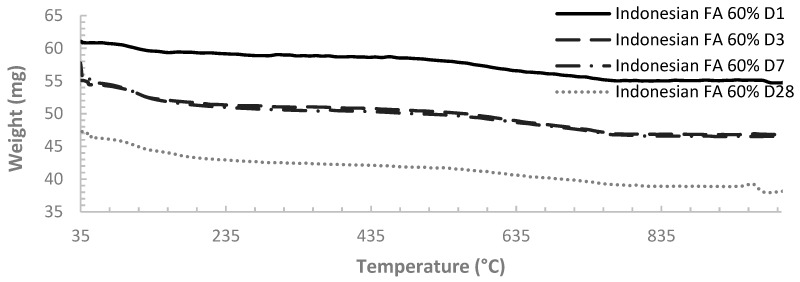
The TG curves for 60% Indonesian fly ash paste at ages of 1, 3, 7 and 28 days.

**Figure A5:**
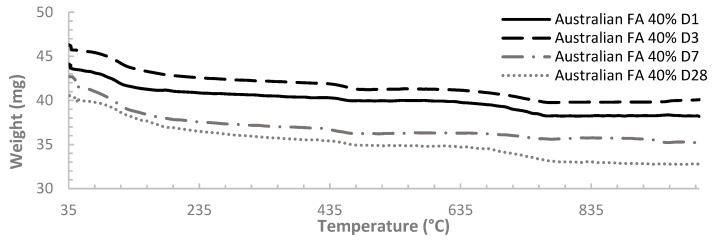
The TG curves for 40% Australian fly ash paste at ages of 1, 3, 7 and 28 days.

**Figure A6:**
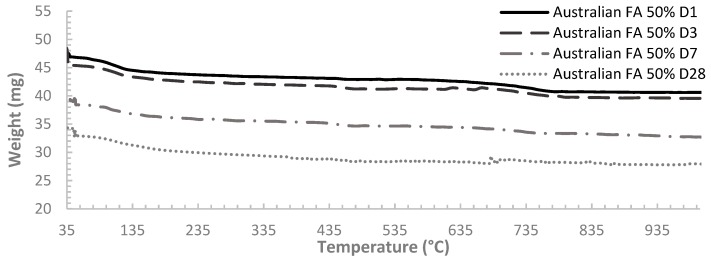
The TG curves for 50% Australian fly ash paste at ages of 1, 3, 7 and 28 days.

**Figure A7:**
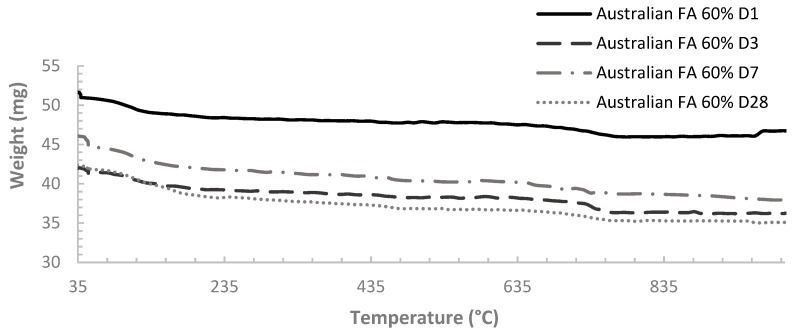
The TG curves for 60% Australian fly ash paste at ages of 1, 3, 7 and 28 days.

## Appendix A

The Thermogravimetric curves for the calculation of W_B_ are show in Figure A1, Figure A2, Figure A3, Figure A4, Figure A5 and Figure A6.
